# Supplementary material for: The nonlinearity of regulation in biological networks
Source: NPJ Syst Biol Appl. 2023 Apr 4;9:10. doi: 10.1038/s41540-023-00273-w (PMC10073134; doi:10.1038/s41540-023-00273-w)
Supplement: Supplementary file 1 — Supplementary Material [file 41540_2023_273_MOESM1_ESM.pdf]

Supplementary Information

The nonlinearity of regulation in biological networks

Santosh Manicka, Kathleen Johnson, Michael Levin and David Murrugarra

Supplementary Table 1: Dataset Description

**Pubmed** ID	**Description**	**Category 1**	**Category 2**	**Linear Approximability**
18852469	A model describing the survival of cytotoxic T lymphocytes in Tcell large granular lymphocyte (T-LGL) leukemia	Disease	Metazoan	80.28235
22102804	A model of Tcell large granular lymphocyte (T-LGL) leukemia	Disease	Cancer	79.27166
26102287	A model describing the infection response of Clostridium difficile to antibiotic treatment	Disease	Primitive	75.69523
23169817	A model of the simplified p53 network with low DNA damage	Disease	Cancer	72.36818
24564942	A threshold model of the C. elegans early embryonic cell-cycle	Regulatory	Metazoan	62.13963
23169817	A model of the simplified p53 network with high DNA damage	Disease	Cancer	59.89628
28209158	A model of tumorigenic transformation of human epithelial cells	Disease	Cancer	57.49518
30104572	A model of cell state transition of epithelial-to-mesenchymal transition (EMT)	Disease	Cancer	56.90066
25189528	A model of epithelial-to-mesenchymal transition (EMT) integrating the signaling pathways involved in developmental EMT and known dysregulations in invasive hepatocellular carcinoma (HCC)	Disease	Metazoan	56.86491
29632237	A model used to identify key protiens regulating bortezomib	Disease	Cancer	55.24462
31516637	A model of cellularmetabolic flexibility where The inhibitor, Pyruvate, only blocks PDKactivity when at least one of its activators is absent from the system.	Metabolic	Metazoan	54.61028
19185585	A model of budding yeast cell-cycle	Regulatory	Primitive	54.46379
27148350	A model linking the nerve growth factor NGF to the proliferation or differentiation outcomes of the cell	Biochemical	Metazoan	52.98308
23081726	A model of the Influenza A virus replication cycle	Disease	Primitive	52.82458
26573569	A regulatory network model of the differentiation of the human gonadal cells into testes or ovaries	Regulatory	Metazoan	52.75945

31516637	A model of cellularmetabolic flexibility where the inhibitor, Pyruvate, always blocks PDKactivity when it is present in the system.	Metabolic	Metazoan	52.21167
30038409	A model of genes/proteins responsible for loss/gain offunction in human pluripotent stem cells	Regulatory	Metazoan	51.17677
24970389	A protein interaction model to elucidate drug resistance in breast cancer	Disease	Cancer	50.66349
22102804	A reduced model of Tcell large granular lymphocyte (T-LGL) leukemia	Disease	Metazoan	50.23742
31130988	FA-CHKREC (G2 checkpoint and the checkpoint recovery) model, which explores how FA cells might use DDA (DNA damage adaptation)	Disease	Metazoan	45.55726
32054948	A model of the G1/S checkpoint regulation contemplating the regulatory influences of both miR-34a and miR-16	Disease	Metazoan	45.41591
16542429	A model of the regulatory network governing the differentiation of CD4+ T cells	Biochemical	Metazoan	44.35813
26244885	A model of the Septation Initiation Network that controls cytokinesis in fission yeast	Regulatory	Metazoan	44.00536
22253585	A model of host immune response to single and coinfection	Disease	Primitive	43.39992
30024932	A model of the G2/M checkpoint arrest regulation contemplating the influence of miR-449a	Disease	Metazoan	41.20924
29378814	a model to predict stabilized Pluripotent stem cells (PSC)gene regulatory network (GRN) states in response to input signals	Regulatory	Metazoan	39.44636
28584084	A model encompassing the main transcription factors and signaling components involved in myeloid and lymphoid development.	Biochemical	Metazoan	38.83446
24970389	A protein interaction model to elucidate drug resistance in breast cancer	Disease	Cancer	38.59772
19524598	The simplified model of regulation of apoptosis via the NFkB pathway	Biochemical	Metazoan	37.31991
22448278	A model of Mycobacterium tuberculosis	Disease	Primitive	35.75686
24970389	A cell signaling network model to elucidate drug resistance in breast cancer	Disease	Cancer	34.81927
30953496	A model integrating the insulin resistance pathway with pancreatic β-cell apoptosis pathway which are responsible for Type 2 diabetes mellitus (T2DM)	Disease	Metazoan	34.81698
24970389	A protein interaction model to elucidate drug resistance in breast cancer	Disease	Cancer	34.08386

31949240	A model of renovascular disease (RVD)	Disease	Metazoan	33.98901
28426669	A model of the gene regulatory network Arabidopsis thaliana	Regulatory	Plants	33.95125
28639170	A model of the wellknown arabinose operon in E. coli	Metabolic	Primitive	33.72184
21853041	A model of myeloid differentiation	Regulatory	Metazoan	32.63689
30953496	A model integrating the insulin resistance pathway with pancreatic β-cell apoptosis pathway which are responsible for Type 2 diabetes mellitus (T2DM)	Disease	Metazoan	32.06319
28381275	A model of colorectal tumorigenesis	Disease	Cancer	32.06171
26616283	A model of oncogenic pathways in NB	Disease	Cancer	31.66786
26340681	A model of cell-cycle regulation in plants	Regulatory	Plants	31.66438
23868318	A model of FGF signaling pathway in drosophila	Regulatory	Metazoan	31.52491
25908096	A model of iron acquisition and oxidative stress response in the fungal pathogen aspergillus fumigatus	Metabolic	Primitive	31.51053
22267503	A model of Fanconi Anemia/Breast Cancer pathway elucidating the repair of DNA strands	Disease	Metazoan	31.23094
26244885	A model of the Septation Initiation Network	Regulatory	Metazoan	31.0255
27542373	A model of the allowed long-term behaviors of the stomatal opening process in plants	Signaling	Plants	30.77432
28426669	A model of the gene regulatory network Arabidopsis thaliana	Regulatory	Plants	30.44945
24970389	A protein interaction model to elucidate drug resistance in breast cancer	Disease	Cancer	30.28947
31516637	A model of cellular metabolic flexibility where Fatty Acid mediated activation of PDK bypasses Pyruvate mediated inhibition.	Metabolic	Metazoan	29.3459
29237040	A logical model of initiation of the metastatic process in cancer	Disease	Cancer	28.56148
16464248	A model of the Tcell receptor signaling	Signaling	Metazoan	27.97508
24970389	A protein interaction model to elucidate drug resistance in breast cancer	Disease	Cancer	27.57258
22253585	A model of host immune response to single and coinfection	Disease	Primitive	27.4484
22192526	A model of the Arabidopsis thaliana root stemcell niche	Signaling	Plants	27.19403
29230182	A model of the molecular regulatory network involved in the control of angiogenesis	Biochemical	Metazoan	26.51007
30116195	A model of the regulation in the differentiation process of major Tcell subtypes, i.e., Th1, Th2, Th17 and iTreg cells.	Biochemical	Metazoan	26.25546

23743337	A gene regulatory network model of T lymphocyte differentiation	Regulatory	Metazoan	26.18992
24079299	A regulatory model of Salmonella typhimurium	Disease	Primitive	25.50749
28187161	A Boolean network CARENET (CAmbium REgulation gene NETwork) for modeling cambium activity	Regulatory	Plants	24.49806
26207376	A model of gene regulation in the cardiac progenitor cells involved in early vertebrate development	Regulatory	Metazoan	23.17666
27464342	A model of the M1 (LPS-activated) and M2 (IL-4/13-activated) macrophage polarization types	Signaling	Metazoan	22.47601
11082279	A model of the ras,Äìraf,ÄìMAPK/ERK signaling pathway	Biochemical	Metazoan	22.18949
24250280	A comprehensive and generic reaction map for the MAPK signaling network	Disease	Metazoan	21.58656
29622038	A phenotype control kernel (PCK) model of the mitogen-activated protein kinase(MAPK) model	Disease	Metazoan	21.54371
26385365	A model of how mutations in the FA/BRCA pathway cause Fanconi anemia	Disease	Metazoan	21.48794
22253585	A model of within-host immunodynamics	Disease	Primitive	20.64292
26408858	A model of the regulatory network of lymphopoiesis	Regulatory	Metazoan	20.25874
24376455	A model of a metastatic melanoma network	Disease	Cancer	20.25273
24069138	A threshold model of the cell-cycle control network of yeast S. pombe	Regulatory	Primitive	20.16704
26751566	A dynamic model for the regulatory network that controls terminal B cell differentiation	Biochemical	Metazoan	19.476
25538703	A model elucidating the role of the CAV1 scaffold protein in Tcell leukemia	Disease	Metazoan	19.43595
31516637	A model of cellularmetabolic flexibility where Acetyl-CoA mediated activation of PDK bypasses Pyruvate mediated inhibition.	Metabolic	Metazoan	19.41784
29186334	A model to describe temporal expression patterns observed in GMP-derived cells	Regulatory	Metazoan	18.79806
1753781	A model of tumor immunity	Disease	Cancer	18.37732
23868318	A model of receptor signaling pathways, including EGFR, G-protein-coupled receptor, integrin, and stress pathways	Regulatory	Metazoan	16.81161

17722974	A model of Tcell activation via the Tcell receptor, the CD4/CD8 co-receptors, and the accessory signaling receptor CD28	Signaling	Metazoan	16.73999
30281473	A model of the aberrant signaling in pancreatic cancer	Disease	Cancer	16.02394
20169167	A model of the Pseudomonas syringae	Disease	Primitive	15.87294
23056457	A model of gene regulation underlying early cardiac development in mammals	Regulatory	Metazoan	15.83555
28361666	A model of castration-resistant prostate cancer	Disease	Cancer	15.29735
27138333	A model of the signaling network associated with RCP-driven invasive migration	Disease	Cancer	15.04079
16968132	A model of water conservation via stomatal opening in plants	Signaling	Plants	13.58676
20221256	A model of the interplay between the NFkB pro-survival pathway, RIP1-dependent necrosis, and the apoptosis pathway in response to death receptor-mediated signals	Biochemical	Metazoan	13.55924
23233838	A signaling pathway model of apoptosis in yeast	Regulatory	Primitive	12.98085
29596489	A mathematical in silico model that robustly recapitulates the crosstalk between IGF and Wnt signaling.	Signaling	Metazoan	12.92163
22962472	A model of keratinocyte cell migration mediated by hepatocyte growth factors	Signaling	Metazoan	12.1657
27613445	A model of the signaling pathways that control S-phase entry and a specific-type of senescence called geroconversion	Regulatory	Metazoan	10.90313
18463633	A model of cyclic gene expression during cell division in budding yeast	Regulatory	Primitive	10.87729
25063553	A model of the hormonal control of hepatic metabolism using insulin and glucagon signaling	Metabolic	Metazoan	10.86576
25063553	A model of the hormonal control of hepatic metabolism using insulin and glucagon signaling	Metabolic	Metazoan	9.87147
23868318	A model of receptor signaling pathways, including EGFR, G-protein-coupled receptor, integrin, and stress pathways	Regulatory	Metazoan	9.859414
20221256	A model of apoptosis involving death-receptor mediated signals	Regulatory	Metazoan	9.835997
25163068	A model of the complete quorum sensing system of Pseudomonas aeruginosa	Signaling	Primitive	9.655856
19422837	A model of apoptosis involving external growth factors	Signaling	Metazoan	9.007914
16873462	A model of the mammalian cell-cycle	Regulatory	Metazoan	8.110625
30518777	A model of the ABA signaling pathway	Signaling	Plants	7.068097

25780058	A model of a Rho-family GTPases signaling network	Disease	Metazoan	6.516515
25063553	A model of the hormonal control of hepatic metabolism using insulin and glucagon signaling	Metabolic	Metazoan	6.489934
25063553	A model of the hormonal control of hepatic metabolism using insulin and glucagon signaling	Metabolic	Metazoan	6.431252
26163548	A signaling pathway model describing the interaction between a drug and cellular outcomes in multiple myeloma cells	Signaling	Metazoan	5.942066
29206223	A model to predict different in-silico knockouts that prevent key SASP-mediators, IL-6 and IL-8, from getting activated upon DNA damage	Biochemical	Metazoan	5.414375
26446703	A model of colitis-associated colon cancer involving P53, MDM2, and AKT	Disease	Metazoan	4.918872
19144179	A model of glucose regulation in yeast	Metabolic	Primitive	4.804903
30530226	A model of the crosstalk between the TGF-β,p38 MAPK and cell-cycle checkpoint pathways which qualitatively describes this dual behavior of TGF-β	Signaling	Metazoan	4.597779
21563979	A model of glucose regulation via the Lac operon	Metabolic	Primitive	3.447058
23049686	A model of cell-cycle in budding yeast	Regulatory	Primitive	3.288717
23868318	A model of receptor signaling pathways, including EGFR, G-protein-coupled receptor, integrin, and stress pathways	Regulatory	Metazoan	2.027278
17010384	A model used to simulate a molecular pathway between two neurotransmitter receptors: dopamine and glutamate receptors	Signaling	Metazoan	0.686516
23171249	A regulatory model of the TOL multiprotein complex system found on the cell membrane of gram negative bacteria	Metabolic	Metazoan	0.508221
23868318	A model of interaction between nine key signaling pathways in drosophila	Regulatory	Metazoan	0.074953
16464248	A model of a typical signaling pathway where mass and signal flow occur simultaneosly	Signaling	Metazoan	0
18194572	A logical model of HGF and H. pylori induced c-Met signal transduction	Disease	Metazoan	0
27594840	A model linking the hematopoietic stem progenitor cells and mesenchymal stromal cells in the bone marrow	Disease	Metazoan	-0.19119
23520449	A model of body segmentation in drosophila melanogaster	Biochemical	Metazoan	-0.30788

23764028	A model of the effect of TGF-b1 on misquitos with the parasite Plasmodium falciparum	Disease	Metazoan	-0.71147
21639591	A model for phospholipase C-coupled calcium signaling pathways	Signaling	Metazoan	-0.76057
18433497	A model of cellular immune response integrating toll-like receptor, interferon, NF-kappaB and apoptotic pathways	Signaling	Metazoan	-1.00938
19025648	A model of physiological regulation in cholestrol biosynthesis	Metabolic	Metazoan	-1.24445
30546316	A model for the generegulation driving macrophage polarization to the M1, M2a, M2b, and M2c phenotypes	Signaling	Metazoan	-2.01878
23469179	A model of cancer network regulated by miR-17-92 cluster	Disease	Cancer	-2.71715
19118495	A regulatory model of the cell-cycle involving the transmembrane tyrosine kinase ERBB2	Disease	Metazoan	-3.46556
23134720	A model linking the oxidative stress pathway to apoptosis	Biochemical	Metazoan	-3.86379
26346668	A model is used to map environmental and flow induced signals to endothelial cell phenotype (proliferation,migration, apoptosis, and lumen formation)	Disease	Cancer	-4.09752
32870080	A model of the lac operon with feedback	Regulatory	Primitive	-4.53977
26090929	An integrated regulatory and signaling pathway model of the fate of the CD4+ T immune cells	Biochemical	Metazoan	-6.53616
20659480	ABA-induced closure model (ABA signal transduction network)	Signaling	Plants	-9.87253
25980672	A model of the melanogenesis signaling network	Signaling	Metazoan	-9.90163
21968890	A model of signaling pathway involving the pro-inflammatory cytokines interleukin 1 (IL-1)	Signaling	Metazoan	-13.6196
31048917	A model of Drosophila melanogaster	Regulatory	Metazoan	-13.6436
23868318	A model of interaction between nine key signaling pathways in drosophila	Regulatory	Metazoan	-13.9899
21968890	A model of signaling pathway involving the pro-inflammatory cytokines interleukin 6 (IL-6)	Signaling	Metazoan	-14.0946
19622164	A model of the external signaling molecule TNFa	Disease	Metazoan	-17.2082
19622164	A model of the external signaling molecule TNFb1	Disease	Metazoan	-17.4249
28455685	A model of the epidermal growth factor receptor signaling of a breast epithelial cell line, MFC10A.	Disease	Cancer	-18.4117

30323768	A model gene regulatory network (GRN) of latent proviruses in resting CD4+ Tcell to visualize thecomplexity of the HIV-1 gene expression	Disease	Metazoan	-20.8165
26528548	A regulatory network model of early metasis development	Disease	Cancer	-78.6659
20862356	A gene regulatory model of spatial expression patterns in the cerebral cortex	Regulatory	Metazoan	-92.6647
